# Evaluating the successful implementation of evidence into practice using the PARiHS framework: theoretical and practical challenges

**DOI:** 10.1186/1748-5908-3-1

**Published:** 2008-01-07

**Authors:** Alison L Kitson, Jo Rycroft-Malone, Gill Harvey, Brendan McCormack, Kate Seers, Angie Titchen

**Affiliations:** 1 Green College, University of Oxford, Woodstock Road, Oxford OX2 6HG, UK; 2 Centre for Health Related Research, School for Health Care Sciences, College of Health & Behavioural Sciences, University of Wales, Bangor, UK; 3 Centre for Public Policy and Management, Manchester Business School, University of Manchester, Booth Street West, Manchester M15 6PB, UK; 4 Institute of Nursing Research, University of Ulster, Shore Road, Newtownabbey, Co. Antrim, BT37 0QB, Northern Ireland, UK; 5 RCN Institute, School of Health and Social Studies, University of Warwick, Coventry CV4 7 AL, UK; 6 Fontys University of Applied Science, Eindhoven, The Netherlands

## Abstract

**Background:**

The PARiHS framework (Promoting Action on Research Implementation in Health Services) has proved to be a useful practical and conceptual heuristic for many researchers and practitioners in framing their research or knowledge translation endeavours. However, as a conceptual framework it still remains untested and therefore its contribution to the overall development and testing of theory in the field of implementation science is largely unquantified.

**Discussion:**

This being the case, the paper provides an integrated summary of our conceptual and theoretical thinking so far and introduces a typology (derived from social policy analysis) used to distinguish between the terms conceptual framework, theory and model – important definitional and conceptual issues in trying to refine theoretical and methodological approaches to knowledge translation.

Secondly, the paper describes the next phase of our work, in particular concentrating on the conceptual thinking and mapping that has led to the generation of the hypothesis that the PARiHS framework is best utilised as a two-stage process: as a preliminary (diagnostic and evaluative) measure of the elements and sub-elements of evidence (E) and context (C), and then using the aggregated data from these measures to determine the most appropriate facilitation method. The exact nature of the intervention is thus determined by the specific actors in the specific context at a specific time and place.

In the process of refining this next phase of our work, we have had to consider the wider issues around the use of theories to inform and shape our research activity; the ongoing challenges of developing robust and sensitive measures; facilitation as an intervention for getting research into practice; and finally to note how the current debates around evidence into practice are adopting wider notions that fit innovations more generally.

**Summary:**

The paper concludes by suggesting that the future direction of the work on the PARiHS framework is to develop a two-stage diagnostic and evaluative approach, where the intervention is shaped and moulded by the information gathered about the specific situation and from participating stakeholders. In order to expedite the generation of new evidence and testing of emerging theories, we suggest the formation of an international research implementation science collaborative that can systematically collect and analyse experiences of using and testing the PARiHS framework and similar conceptual and theoretical approaches.

We also recommend further refinement of the definitions around conceptual framework, theory, and model, suggesting a wider discussion that embraces multiple epistemological and ontological perspectives.

## Background

The spread of best practice and the use of best evidence remain sporadic. There continues to be a tension between policy imperatives and the ability to successfully support and enable local developments. Arguably the debate about how to implement evidence effectively reflects a lack of a true appreciation or understanding of the multiple factors involved. However, there has been a shift away from the traditional notion that getting evidence into practice is straightforward. Until relatively recently the spread of evidence was seen as a linear and technical process at the level of the individual, and was described as changes in clinicians' behaviour in line with evidence-based guidelines [1]. Now there is widespread recognition that guideline implementation, and evidence implementation more generally, requires whole system change implicating both the individual and organisation ([2,3]). Despite a growing awareness that getting evidence into practice is a complex, multi-faceted process, there remains a lack of knowledge about what methods and approaches are effective, with whom and in what contexts.

The PARiHS framework represents the complexities of implementing evidence into practice. Previous papers have reported on the development of the framework over time [4-9]. Other authors have also reported on their use of PARiHS as a theoretical and practical heuristic to guide research and practice development work [10-14]. This paper integrates our work to date and presents the hypothesis that the PARiHS framework could be applied by practitioners as a diagnostic and evaluative tool to successfully implement evidence into practice, and by practitioners and researchers to evaluate such activity. In addition, the current and future challenges in relation to the PARiHS framework and to the field more generally are identified and discussed.

### The PARiHS framework – an overview

Within the PARiHS framework, successful implementation (SI) is represented as a function (f) of the nature and type of evidence (E), the qualities of the context (C) in which the evidence is being introduced, and the way the process is facilitated (F); SI = f (E, C, F). Detailed descriptions exist in the literature on the development and empirical evaluation of the PARiHS framework [4-9]. The framework has been refined through two phases of research and development and is currently in its third or current phase (see Table 1 for a comprehensive summary). The unique characteristic of the PARiHS framework was that it proposed a three-dimensional framework within which to interpret successful implementation, arguing that elements could be located on a continuum of "high" to "low" evidence and context

**Table 1:** Summary of development and refinement steps of PARiHS framework

**Phase 1: Development and Concept Analysis 1998 – 2002**	
**Origins**	- Emerged from working with clinicians in helping them to improve practice, introduce new ideas and implement guidelines.
**Main Attributes**	- Successful implementation of new ideas (evidence, guidelines, etc.) is a function of the interrelations between three key elements – evidence, context, facilitation: SI = f (E, C, F)
**Face Validity**	- 4 research studies were analysed retrospectively to test the hypothesis that SI = f (E, C, F). - Strong face validity.
**Construct Validity**	- Assumption that Evidence, Context and Facilitation as described are discrete and interdependent and can be manipulated in a purposeful way.
**Refinement**	- Need to undertake detailed concept analysis of each of the elements and sub-elements (E, C, F).
**Future Action**	- Concept analysis and empirical testing.
**Publications**	- [4-7]

**Phase 2: Empirical Case Studies 2001–2003**	

**Main Research Questions**	- What factors do practitioners identify as the most important in enabling implementation of evidence into practice? - Do concepts of evidence, context and facilitation constitute the key elements of a framework for getting research into practice?
**Refinement**	- Important additions to evidence – information from local context; resources, physical and political influences in context. - Experience of facilitators on the ground with very little, limited support.
**Future Action**	- Further testing through larger scale empirical enquiry testing the checklist and developing an evaluation tool.
**Publications**	- [5, 8]

**Phase 3: Development of Diagnostic/Evaluation Tool 2003 – Present**	

**Main Research Questions**	- Is it possible to develop a diagnostic and evaluative tool to measure the successful implementation of new ideas (evidence, innovation) into practice using the PARiHS framework?
**Refinement**	- Pre-test diagnostic phase
	• Summary scores for evidence and context (E, C)
	• Narrative summary
	• Information on prototypes of facilitation approaches
	- The facilitation process
	- The post test evaluation
	• Re-plot summary scores for E + C
	• Narrative summary
	• Evaluation of facilitation approach

### Summary of the development and refinement steps for PARiHS framework. (See Table 1)

The main features and assumptions of the framework are:

1. Evidence encompasses codified and non-codified sources of knowledge, including research evidence, clinical experience including professional craft knowledge, patient preferences and experiences, and local information.

2. Melding and implementing such evidence in practice involves negotiation and developing a shared understanding about the benefits, disbenefits, risks, and advantages of the new over the old. This is a dialectical process that requires careful management and choreography, and one that is not done in isolation; in other words, it is a team effort.

3. Some contexts are more conducive to the successful implementation of evidence into practice than others – these include contexts that have transformational leaders, features of learning organisations, and appropriate monitoring, evaluative, and feedback mechanisms.

4. There is an emphasis on the need for appropriate facilitation to improve the likelihood of success. The type of facilitation, and the role and skill of the facilitator that is required is determined by the "state of preparedness" of an individual or team, in terms of their acceptance and understanding of evidence, the receptivity of their place of work or context in terms of the resources, culture and values, leadership style, and evaluation activity. Facilitators work with individuals and teams to enhance the process of implementation.

The objective of the current phase of our work is to build on the concept analysis and clarification undertaken in phases one and two, and to evaluate the current framework through the development and testing of diagnostic and evaluative instruments to assist in the process of knowledge translation. Whilst conducting this phase, a number of challenges have arisen, which, whilst reflecting the particular complexities of the PARiHS framework's development, are also relevant to current debates in the field of knowledge translation. These include:

1. Understanding how the conceptual framework relates to, and informs the development of, integrated theoretical positions that are practically useful and theoretically robust.

2. Engaging in the challenges measurement presents and particularly within a theoretical position which argues that the intervention (a precise and tailor-made type of facilitation constructed by a skilled facilitator and those involved in the implementation process) is contingent upon the diagnosis of the evidence and context elements and clarifying facilitation as an intervention.

These issues will be considered in turn.

## Discussion

### Conceptual frameworks, theories, and models of knowledge translation: seeking greater clarity

There is a growing interest in the literature around clarification of terminology used in implementation science and also around the use of such mental devices as conceptual frameworks, theories, and models [15]. However, despite the debate there is little consistency in the way these terms are used and in particular there is a tendency to substitute one term for another without due consideration of the deeper meaning attributed to such terms. For example, one of the earliest conceptual frameworks developed by Havelock and colleagues [16] was derived from Roger's Diffusion of Innovation Theory [17]. Their Research Dissemination Utilization Conceptual Framework was built upon two key components – knowledge building and institutionalizing. Knowledge building (or synthesis) would integrate theories to replace fragmented approaches, and their concept of institutionalisation enabled the new knowledge to be transferred through integrated, cross discipline, cross boundary programmes. Of primary importance is the relationship of trust and mutual co-operation that needs to be built up between researchers, policy makers, decision makers, and practitioners.

Greenhalgh *et al.*'s synthesis of the literature on diffusion of innovations has also produced a conceptual map [18]. Described by the authors as a "conceptual model" it is not expected to be used in any practical way to guide actions. Rather, it is a mental representation of the many elements that need to be considered. Each element was derived from rigorous review of the literature, and the antecedent theories informing (and shaping) the elements are described. For example, one element of the model is diffusion, and within that element several approaches to diffusion are described – social networks, marketing, expert opinion – all aspects/actions of a wider theoretical perspective.

In contrast, Graham *et al*. have offered their own conceptual framework to help elucidate what they believe to be the key elements of the knowledge-to-action process [15]. Essentially the framework is divided into two elements or concepts: knowledge creation and action, with specific steps within each element. (For a detailed explanation see Graham *et al*. [15].) The framework also begins to articulate the embedded theoretical positions that determine action. Graham and colleagues are particularly interested in theories of planned action [19] and have identified over 60 theories or frameworks (although the authors do not distinguish between these terms in their paper).

The PARiHS framework shares similar characteristics to the above conceptual frameworks – the identification of elements and relationships, embedded (either explicit or implicit) theoretical positions, and a way of trying to explain a complex set of phenomena that enable action to be taken. What distinguishes the PARiHS conceptual framework from the others is that as well as mapping the interrelationships, PARiHS has the potential to be used as a practical and pragmatic tool by practitioners and researchers at the local level. This is the claim, and it is also the hypothesis that we are continually testing.

In addition to the literature on conceptual frameworks, there is an emerging debate about theory use and development in knowledge translation work [20-22]. Theory use is presented by its supporters as a promising approach to better understanding the 'black box' of implementation [23]. Estabrooks *et al.*'s description illustrates the vast array of theories that could be used to determine or explain the process of implementation, and also demonstrates the lack of agreement on terms – models, frameworks, and theories are used interchangeably [20]. What seems to be emerging is that the term theory is used when a set of relationships are explained and there is some predictive capability: for example, the use of social influence strategies to introduce clinical guidelines [24]. The use of the term model seems much more diffuse – ranging from prescriptions on how to implement research into practice [25-27] to more specific descriptions of a theoretical perspective, e.g., Prochaska *et al.*'s transtheoretical model of health behaviour change [28]. However, to date, the prevalent view about theory and knowledge translation has tended to focus upon positivistic interpretations that favour deterministic explanations [23].

### One typological definition

Therefore, one important question is whether it matters what we call these mental devices. Is there a difference between conceptual frameworks, theories, and models, and, if so, what and how would such differentiations help our understanding of the complex world of research implementation or knowledge translation? Identical questions have been posed in the discipline of public policy analysis and implementation, as well as theory development [29]. The policy world is complex, with multiple elements interacting over time. How can complex situations be simplified in order to understand them, and how can the tension be managed between the exploration of specific interventions within a system and the overall appreciation of the impact of the intervention on the whole system? In attempting to create a deeper understanding, Sabatier and colleagues have described three dominant approaches to policy analysis and implementation [29]. Within this analytic framework they have also put forward a typology for understanding the different 'mental representations' we could use to hold onto the complex world. This analytic framework, first proposed by Ostrom [30,31], has been used as a way of trying to make sense of the different ways that frameworks, theories, and models could be used to inform our research activity. Ostrom [31] argued that:

"...given the need for multiple disciplinary languages and given the multiple levels of analysis involved in studying configural relationships between rules, relevant aspects of the world and cultural phenomena, the study of institutions does depend on theoretical work undertaken at three levels, namely frameworks, theories and models"

Both Sabatier [30] and Ostrom [31] argue that for the effective development of policy theory the following distinctions can be made. A conceptual framework identifies a set of variables and relationships that should be examined in order to explain the phenomena. Indeed, a framework can provide anything from a skeletal set of variables to something as extensive as a paradigm, where a paradigm is the notion which places emphasis on professional consensus within a particular scientific community. It stands for the entire constellation of beliefs, values, and techniques shared by members of that community [32]. A conceptual framework need not specify the direction of relationships or identify critical hypotheses. In contrast, a theory provides a denser and logically coherent set of relationships. Theories can offer views on the causal relationships and seek to explain the phenomena, although from an interpretative perspective theories also play a vital role in offering explanations rather than causal relationships [33]. Numerous theories may be consistent within the same framework. Models, by contrast, represent a specific situation, are narrower in scope, and are more precise in their assumptions [29,31]This approach would seem to offer one way of testing conceptual coherence between the typological levels within the discourse of implementation science.

For Ostrom [31], a conceptual framework helps to identify elements and relations among those elements that one needs to consider for an analysis of organisations (at multiple levels of operation – individual, team, unit, and whole systems level) and their ability to absorb and adopt innovations. Frameworks also organise diagnostic and prescriptive enquiry and provide a more general list of variables that can be used to analyse types of institutional arrangements. Conceptual frameworks provide a meta-theoretical language that can be used to compare theories, and they attempt to identify universal elements of any theory relevant to the same kind of phenomena that would need to be included in order to understand the "bigger conceptual picture". Thus, for example, in Ostrom's analysis, the question would be whether the elements as identified in the PARiHS framework survive continuous scrutiny and testing against multiple theories at multiple levels within the organisation that have a relevance and coherence to research implementation strategies. So long as this is the case, the elements remain intact: once exceptions begin to emerge, the basic tenets of the conceptual framework are placed under further scrutiny.

Whilst the PARiHS framework has been subject to an ongoing development process, questions about it remain, including:

1. How do the elements (evidence, context, and facilitation) and sub-elements interrelate and interact with each other and across the different layers of the organisation?

2. Do the elements and sub-elements have equal weighting in getting evidence into practice?

3. Is the content of the framework comprehensive?

For the framework to usefully inform the development and testing of current and emerging theories, these questions need to be answered. Arguably, work to date has provided evidence of the framework's content and construct validity [6-8,11]; that is, we can be reasonably confident that PARIiHS is a conceptually robust framework. This is a sufficient basis upon which to begin testing a range of theories and building new theories inductively and deductively. The test of its effectiveness as a conceptual framework is whether it can generate such diagnostic, analytic, prescriptive, interpretative, and evaluative discourse.

According to Ostrom [31], key questions to test the coherence of any conceptual framework, include:

1. Does the framework provide a coherent language for identifying universal elements of theories attempting to explain an important range of phenomena?

2. Does the framework help scholars to identify similarities or differences of diverse theories as well as to analyse the relative strengths and weaknesses of theories in explaining particular types of phenomena?

3. Does the framework stimulate new theoretical developments?

Questions used to test the usefulness of any conceptual framework in empirical research include:

1. Does the framework help organise empirical research in those areas where well-specified theories are not yet formulated?

2. Does empirical research drawing in the framework lead to new discoveries and better explanation of important phenomena?

3. Can the framework be applied to multiple levels of analysis in empirical research?

And finally, in relation to conceptual frameworks, Ostrom's typology includes questions about the ease by which the framework aids the better understanding and dialogue across disciplinary boundaries:

1. Does the framework encourage integration across other disciplines?

2. Is the framework consistent with other frameworks initially developed to focus on a particular level of analysis?

3. Does the framework perform better than others in a similar stage of application?

These questions are helpful because they enable an assessment to be made of the PARiHS framework's stage of development and provide an agenda for further work. For example, there is evidence to indicate that PARiHS does help organise empirical research where theories are yet to be formulated [11,34], and that the framework has led to better explanations of important phenomena [10,35]. However, consideration still needs to be given to the framework's capability for theory application and development. Questions about the range and diversity of applicable theories still need to be explicated. Adopting Ostrom's typology, which acknowledges multiplicity, it could be argued that rather than placing PARiHS within one particular theoretical perspective or offering a single theory for research implementation, which could limit its applicability, the framework could be populated by multiple theories, at multiple levels. From this perspective the PARiHS framework would operate similarly to other frameworks outlined earlier, e.g., Graham *et al.*'s [15] Knowledge to Action (KTA) framework that focuses on planned action theories, or Greenhalgh *et al.*'s [18] conceptual model summarising the range of theories that influence diffusion of innovations.

Further consideration of these issues forms the basis of the next phase of work/development of PARiHS and in particular attempts to answer the key questions raised by Ostrom. These include whether the framework can provide a coherent language for identifying universal elements of theories attempting to explain an important range of phenomena, or indeed whether PARiHS can be applied to multiple levels of analysis – such as individual, team, unit, and organisational-wide level?

### Frameworks, theories and models in use: the chess game

How does this analysis help to guide users in successfully implementing evidence into practice? We could use the analogy that the PARiHS framework is like a chess game. There is a defined set of rules and an agreed number of chess players. The pawns, knights, king, queen, bishops, etc. each have a set of rules to follow. Each chess piece has its own provenance or theoretical background that would explain the reason why different pieces move in certain ways. Equally, in each game the unique configuration of the chess pieces creates an almost infinite number of moves that can test the boundaries of movement of each piece, and equally test the boundaries of the higher rules of the game (framework) itself. Each new game could be like a model that will test the theories of the chess pieces within the boundaries of the chess game, *i.e*. the conceptual framework.

However, unlike the chess game, we still do not know the rules (should there be any) of the knowledge translation game and the movements of the different pieces are yet to be fully understood. Of course, this analogy only works if we accept the prior assumption that implementation processes are predictable, and that there are certain causes and effects at work. The converse position is to assume that all interactions are random, and that there is no predictive capacity because of the complexity involved in working with so many variables. Given that we do not know which of these positions is the more accurate, and it is largely dependent on one's world view of how these issues should be studied, we argue that it is legitimate to proceed with the "chess game analogy" until there is sufficient evidence amassed to disprove it. Taking such an *a priori *position is consistent to Kuhn's notion that all good scientific endeavours are about the business of empirically falsifying propositions within a theoretical framework [29].

Thus, to conceptualise the process of introducing evidence into practice, we are suggesting that to use the PARiHS framework, practitioners and researchers contemplate the interplay of evidence, context, and facilitation, as well as their sub-elements. Each element and sub-element has a conceptual and theoretical order that determines its intrinsic properties; the interaction of these elements is conditional on their state, maturity, context, and many other factors. The modelling or experimentation that can be constructed is a way of tracking the nature of the different elements and beginning to map the processes by which change occurs through the interaction of these elements.

Table 2 illustrates how the PARiHS framework elements (evidence, context and facilitation) could draw on multiple theoretical perspectives. This, in turn, offers even more models that can then be used to explore systematically the consequences of these propositions in a clearly defined and controlled set of outcomes. What begins to emerge when looking at Table 2 is that, depending on the theoretical approach taken, there is any number of entry points into testing elements of the framework.

**Table 2:** Conceptual frameworks, theories and models: Interrelationship between the elements of the PARiHS framework and linked theories and models. (based on Ostrom's typology).

	Conceptual Framework	Theory	Model
Definition	Identifies a set of variables and relationships that should be examined in order to understand the phenomenon	Provides a more dense and logically coherent set of relationships and offers views (hypotheses) on the causal relationships and seeks to explain the phenomena	Represents a specific situation; is narrower in scope and more precise in its assumptions
Dimensions EVIDENCE Sub-elements Research Clinical Experience Patient Experience Routine Data	Evidence Evidence is a broad term comprising 4 key elements : research, clinical experience, patient preferences and routine information Melding and implementing evidence involves negotiating and developing shared understandings It is a dialectical process	What theories would inform the way evidence has been conceptualised within the PARiHS framework? E.g. How would decision making theory or clinical reasoning or cognitive theory inform/influence/alter the way we would try to make sense of how practitioners at clinical level adopt and value a new innovation?	Would we classify guideline implementation as one model to be tested within the wider clinical reasoning/knowledge generation theoretical tradition? Are the use of patient narratives, or audit and feedback more examples of models that can test the broader theoretical positions that inform the conceptual framework?
Context Sub-elements Context Culture Leadership Evaluation	Context Comprises 4 broad areas: Context, culture, leadership and evaluation Some contexts are more conducive to the introduction of new ideas/innovations. It is the interplay of the elements and sub-elements that make implementation easier or more difficult Big complex area operating at multiple levels. Important to be able to see the whole picture when changing practice	The theoretical base of understanding organisations, contexts, cultures and innovation is diverse, multifaceted and very complex. What criteria would you use to select the more appropriate theories that would elucidate how the elements of the PARiHS framework interact? How can theories be integrative in order to explain the realities of real world implementation? How would	Testing different learning styles and experimenting with a variety of leadership roles and styles could be part of the range of interventions or models used. Selecting one leadership approach within leadership theory in general would be part of the multiple models and theories being tested within the framework
Facilitation Sub-elements Purpose, Role Skills and Attributes	Facilitation Broad term describing the human support, guidance, learning, coaching offered by a trained facilitator when initial diagnosis of the "readiness" of the individuals, team and context for the introduction of the innovation The purpose can be technical e.g. introducing a discrete method or "holistic" sustaining and enabling personal development and system transformation Method contingent on diagnosis of individual/team understanding/acceptance of evidence and receptiveness for change of context	Facilitation has a strong theoretical base in humanistic psychology, psychoanalytic group theory and adult learning theory. Therapeutic client-centred approaches, experiential learning and self-efficacy theory also contribute to our overall understanding. The question again remains how researchers and practitioners make sense of these underlying theories to help them construct way of changing practice. Constructing a particular programme or mentoring experience, based on psychoanalytic theory will be different from an approach based on adult learning.	Facilitation models can range from "doing for others" to "enabling others". Doing for others covers episodic contact offering practical help using external change agents. Enabling others focuses more on sustaining partnerships, developing individual potential and encouraging self directed learning Doing for would use the following: Project management techniques, technical, marketing skills Enabling others would select methods around co-counselling, clinical reflection, action learning.

How researchers and practitioners "make sense" of the bigger conceptual framework is a fundamental question and an on-going challenge reflecting the complexities involved. The choice of theoretical perspective will necessarily put a boundary around the area of investigation. For example, if we want to investigate the impact of opinion leaders on research implementation using transformational leadership theory, then we will still be left with the job of integrating these findings into the bigger conceptual picture of how research findings get into practice. Holding one piece of the conceptual jigsaw without negating the possible impact of other factors is very important but very difficult to manage. The proposed links are hypothetical and illustrate the conceptual challenges of any nascent discipline.

Such an approach to framework and theory use and development requires researchers to be flexible and holistic. To date, the knowledge translation literature describes theory use and development as a linear and discrete process in line with more traditional scientific thinking and the search for understanding causation [21,22]. Looking to other methodologies that are less concerned with causation and more focused on explanatory understanding and action (such as realist evaluation) [33] may limit reductionism, and provide enlightening findings about the interactions and complexities involved in knowledge translation activity [23]. However, we still need to be mindful of the relationship between the theory and the subsequent methodology and consider their fit with each other and with philosophical perspectives.

An additional set of definitional challenges that Sabatier and Ostrom's typology raises is their definition of models. Ostrom describes models as precise assumptions about a limited set of parameters and variables [31]. Logic, experimentation, and a variety of simulations can be used to explore systematically the consequences of these assumptions in a limited set of outcomes. Multiple models are compatible with most theories and frameworks.

So, for example, in this typology, we could set up an experiment that would test the model of audit and feedback as a precise intervention. The theoretical underpinning of the model could be decision theory or learning organisation theory, both embedded within the bigger conceptual framework of evidence, context, and facilitation. The challenge then is to draw sufficiently cogent paradigmatic boundaries around the framework so that it does not become a catchall of ideas and conjectures. How we do this is where the real scientific discipline comes into focus and where logical coherence and consistency of terms and relationships are set out for scrutiny, and it is where causal processes seek to explain how certain patterns of phenomena have come about. And, of course, this requires the ability to measure the variables under scrutiny. Our deliberations over the years as to how we move the PARiHS framework from a conceptual artefact to becoming a measure of knowledge translation has led us down the path of whether we have to disaggregate the elements because there is no conceptual coherence or whether we dissect the elements in different ways. The notion that the framework becomes a diagnostic and evaluative measure on the evidence and context axes and informs the facilitation or intervention process has been an important development.

### From conceptual framework to measurement and evaluation

Estabrooks *et al*. have outlined the challenges of measuring knowledge utilisation in health care [36]. These include a lack of underpinning theory, construct clarity, measurement theory, psychometric assessment, and a presumption of linearity. Additionally Rich claimed that there tends to be a bias to measure things that are easy to capture [37]. These measurement challenges reflect the general complexity of research implementation. As described above, the purpose of the PARiHS framework is to provide a map to enable others to make sense of this complexity, and the elements that require attention if implementation is more likely to be successful. The next step is to consider whether the PARiHS conceptual framework lends itself to guiding the development of diagnostic and evaluative approaches and instruments, which could be used by both researchers and practitioners. Our emerging hypothesis is that the PARiHS framework will guide measurement development, and there is growing evidence to support this [10-14]. Given that more theoretical work needs to be conducted on the PARiHS framework, these ideas are at an early stage.

To this end, a briefing summary – *PARiHS Framework: stages of refinement *– [See Additional File 1] shows some draft questions, which may begin to facilitate the identification of those elements within 'evidence' and 'context' that require development work, and active intervention(s) ('facilitation') to be successfully introduced within specific implementation projects. The questions developed in the tool could enable individuals and teams to test their appreciation and understanding of evidence, context and facilitation. For example, using the four sub-elements of 'evidence', the tool enables the development of a better understanding of assumptions and perceptions about the research base, how this conflicts with and/or supports clinical experience, professional judgement, and patient preferences, and whether routine information is sufficiently robust to be able to offer data on current practices as well as what needs to change. Similarly, the questions about 'context' encourage an evaluation of the preparedness of the context to embrace and sustain implementation. These questions could be answered individually and/or through a facilitated dialogue where each team members' assumptions, prejudices, views about existing practice, and the proposed change are discussed and debated. Through this process the team would come to an agreed ranking of the 'readiness' of the team to embrace the new practice, evidence, or innovation.

**Figure 1:**
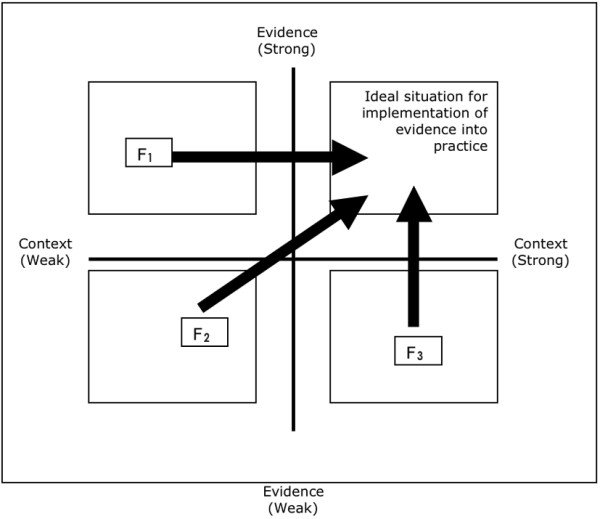
The PARiHS Diagnostic and Evaluative Grid.

We suggest one way of testing this state of readiness is to aggregate responses to the questions, and then translate them onto a grid that plots the position the team judges themselves to be in before they embark upon the implementation process. An example of this is presented in Figure 1 – *The PARiHS diagnostic and evaluative grid*. At this stage, the location on the grid enables an assessment of the type of facilitation support that would most effectively lead to the successful implementation of evidence, likely requiring changes in behaviour and working patterns. The diagnosis identifies the position of the team. The trajectories in Figure 1 illustrate examples of three possible positions:

1. F_1 _= facilitation method for transforming weak context and strong evidence into a highly receptive context

2. F_2 _= facilitation method to manage weak context and weak evidence situation – most challenging and possibly involves issues of safety, basic competence needs to be managed

3. F_3 _= facilitation method to manage strong context and weak evidence situation – issues of routine and power involved

Whilst it is likely that many sources of information will be required to decide on an appropriate course of action, the diagnostic score may provide an indication of the starting point. Facilitation, as the intervention, can then combine a range of approaches ranging from task focused (*e.g.*, project management, resource identification) to more enabling processes (*e.g.*, personal development, action learning). The role of the facilitator then is concerned with assessment of the situation, assessment of individual, team and workplace readiness, development of change and evaluation strategies, support of the implementation process, and coaching and mentoring the team though the change.

The final task is to evaluate and check whether the self-assessment scores have migrated further towards the top right hand quadrant. By considering the evaluation questions, both individuals and teams can evaluate their implementation efforts. These could be administered as both process and summative evaluation opportunities, and progress mapped.

Using PARiHS as the basis for a tool may shift thinking away from conventional, arguably narrow, notions of measurement to more wide ranging and eclectic approaches to evaluation. Given that we predict so much variation in appreciation of evidence and context, the way to test the measurement tool has to accommodate variety and multiple interpretations. Similar messages are coming through from other research teams [15,18,20]. This is why we wish to set up networks of researchers and practitioners who are willing to work together to test out these assumptions and ideas.

### Facilitation as an intervention

The concept of facilitation, defined as "a technique by which one person makes things easier for others", continues to be central to the PARiHS framework [6]. Facilitation is part of a range of roles that have been reviewed in the literature [38,39] which tend to demonstrate that effective implementation of knowledge into practice is a multifaceted process, requires flexibility, and has more to do with the ability to combine a range of different techniques than rigidly prescribing a discrete intervention. From Harvey *et al.*'s concept analysis the following positions have emerged [6]:

1. Facilitation is a process that depends upon the person (the facilitator) carrying out the role with the appropriate skills, personal attributes, and knowledge

2. The purpose of facilitation varies from providing help and support to achieve a goal to enabling individuals and teams to analyse, reflect, and change their own attitudes, behaviours, and ways of working.

3. A "facilitation continuum" has been described, which distinguishes between a "doing for others" role (more discrete, practical, technical and task driven) on the one side to an "enabling and empowering" role which is more developmental, seeking to mentor, guide and support the staff within the system to take control of their own learning and change processes.

4. Facilitation skills are developed through experiential learning [40], and more recently through the acquisition of key facilitation competencies [41].

5. Facilitation as a discrete intervention has been described in the practice development movement in nursing [42-44] and in the quality improvement literature [34].

As a key dimension of the PARiHS framework, facilitation needs to be further refined and tested. Our deliberations have led us to propose that facilitation will be more effective following a diagnosis of the context (C) into which the new knowledge is being introduced and an assessment of practitioners' understanding of and acceptance of the evidence/new knowledge itself (E). The diagnostic should provide data to determine the most appropriate facilitation approach – which, consistent with the evidence, should lead to a multi-faceted, flexible, tailor-made intervention being constructed. Information on individual and teams' understanding of and receptiveness to the new evidence will help determine how much new learning and change is needed. An assessment of the readiness for change from a contextual perspective will cover the PARiHS framework sub- elements of context, culture, leadership, and evaluation [11,14].

The role of the appropriately prepared facilitator, along with the team(s) they are working with, is to construct a programme of change that meets the individual and team's learning needs. The actual ingredients making up this intervention could draw from a whole range of methods – from very task based, planned change programme approaches to much more experiential, action learning approaches.

What we are proposing is consistent with the early observations of Havelock and colleagues, where they argued for greater alignment or linkage between those who generate and implement new evidence and those who are expected to be involved in its application [16]. Equally, Van de Ven and colleagues' work on the management of innovation demonstrates the importance of flexibility and skilled support in achieving successful implementation throughout the length of the implementation process [45]. Also, Greenhalgh *et al*. recommend much greater involvement of practitioners in determining the nature of interventions at local level [18], a perspective that is consistent with the emerging interest in such theoretical perspectives as realistic synthesis [46]. In this interpretivist approach, the research interest in interventions is not so much about their generalisability and standardisation but more about understanding the mechanisms that connect events in a way that changes them (either in the desired direction or in an unexpected way) within a particular context involving particular participants.

What are the next practical steps for developing a series of studies that would begin to test the hypotheses outlined above? First obvious steps are to refine the diagnostic process and associated measures, and the second step is to agree the content of a facilitation training programme that would equip appropriately (and consistently) trained facilitators to work with a number of practice areas wishing to engage in knowledge translation activities.

We argue that more careful theoretical work, modelling, and testing of the concept of facilitation is required because it is the process by which individuals and teams first interact and engage with evidence (either as guidelines, research reports, or any new innovation entering the system) and then try to negotiate its adoption/acceptance into their organisation.

These conclusions would lead one to surmise that the future direction of travel will be around the development of much more complex and bespoke interventions that fit local contexts. Whether this is different from what the PARiHS framework terms "facilitation" is an appropriate question to ask.

### Concluding remarks

There is a small, but growing body of evidence from research and practice that shows the PARiHS framework has conceptual integrity, face and concept validity.

However, there are major challenges ahead if the framework is to help in the systematic exploration of these complexities around the art and science of implementation.

The three challenges outlined include the need to integrate theoretical perspectives into the framework in a way that enables us to make sense of the complexities and to construct appropriate models to explore what works in knowledge translation. PARiHS was developed inductively, which points to an interpretive lens on theory application and development. However, using Ostrom's helpful analytical approach it may be more helpful to begin to see the framework as being populated by various theoretical positions, which some would view as strength, some as a weakness. These issues have yet to be debated in the knowledge translation literature generally, and in relation to the PARiHS framework specifically.

A second area of investigation is the development and testing of diagnostic and evaluative methodologies and associated instruments based on the elements and sub-elements of the PARiHS framework. What seems to be emerging is the need for a high level set of principles (conceptual framework) that can help people on the ground understand what they can do. The principles can offer a framework within which a number of approaches or attempts at implementation and evaluation of the effectiveness of the intervention can be made by both the players on the ground and any researchers involved with them.

Thus, it would seem that the current knowledge base around successful implementation of innovations into practice emphasises processes of engaging local practitioners as well as outlining a set of key principles that help guide the activity.

Whilst there are some studies underway [32], to date there have been few, if any, systematic investigations of facilitation as an intervention. We believe that much more conceptual clarification is needed before the science around it can improve. Equally, the arguments put forward here, that future implementation tools ought to have both diagnostic and evaluative properties also need to be tested. Our proposal to create communities of researchers, practitioners, and other stakeholders undertaking pieces of work to test the whole framework is presented as a way of moving the agenda forward. We see the need for this collaborative approach, not only between researchers but also between research teams and those practitioners at the local level who actually have the task of implementing evidence into practice. This need for greater alignment of these two groups has been reinforced by Greenhalgh *et al.*'s work [18], where they broaden out the notion of implementation of evidence to include innovations in general and by practice development researchers [46]. We also note the semantic and conceptual shift in the discourse and seminal work of Van de Ven and colleagues [47] who had, two decades earlier, tried to measure these very same complexities. Their elegantly designed studies could help us to construct more appropriate studies that take account of the multiple elements at work.

Lastly, we suggested the analogy of a chess game as a way of trying to understand the task in hand. We have a board in front of us (metaphorically speaking) with game pieces (PARiHS elements and sub-elements) whose moves we need to test. Once we know what these pieces do, we can set up the games, *i.e*. the particular interventions, to see what happens. Our research endeavours will, if we are lucky, be able to produce guiding principles for the moves that practitioners can use to successfully implement research into practice. However, we acknowledge the significant work that practitioners will always have to do to transform the principles into effective actions in their own workplaces.

## Summary

1. The PARiHS framework is a useful practical and conceptual heuristic for research implementation but it remains largely untested, hence there is not an evidence base to discount or refine it.

2. The paper summarises the conceptual and theoretical thinking around the use of the framework, inviting colleagues who have or are using it to comment on its utility and effectiveness.

3. The first stages of developing diagnostic and evaluative methodologies based on the framework are presented.

4. Alternative perspectives for thinking about theory use and development are offered.

An implementation science collaborative, working on various elements of the framework is proposed to accelerate the production and testing of its evidence base.

## Competing interests

The author(s) declare that they have no competing interests.

## Authors' contributions

ALK is the lead author and co-ordinator of the paper. JR-M co-wrote and re-drafted the paper. GH, BM, KS, and AT offered ideas contained within the paper, commenting on drafts, reading and approving the final draft of the manuscript. All authors have contributed to the development of the PARiHS Framework, refined the different stages of its development and are responsible for the further refining and testing of the framework.

## Supplementary Material

Additional file 1 **PARiHS Framework: Stages of Refinement**. A table outlining the development of the diagnostic and evaluative measures Click here for file
