# Supplementary material for: Evaluating the successful implementation of evidence into practice using the PARiHS framework: theoretical and practical challenges
Source: Implement Sci. 2008 Jan 7;3:1. doi: 10.1186/1748-5908-3-1 (PMC2235887; doi:10.1186/1748-5908-3-1)
Supplement: Additional file 1 — PARiHS Framework: Stages of Refinement. A table outlining the development of the diagnostic and evaluative measures [file 1748-5908-3-1-S1.doc]

# PARIHS Framework: Stages of Refinement

| Phase 1 – development and concept analysis | Phase 2 – empirical case studies: what factors do practitioners identify as the most important in enabling them to implement evidence into practice? | | Phase 3 – developing a diagnostic and evaluative tool to enable practitioners and researchers to measure Evidence and Context. | |
| --- | --- | --- | --- | --- |
| EVIDENCE | **EVIDENCE: dimensions**  **viewed to be important**  **to accepting new**  **evidence into practice** | | | EVIDENCE: Diagnostic/evaluative question to be answered by clinical leader/team |
| Research – sub-element of evidence | | | | |
| • randomised controlled trials | • well conceived, designed and executed research appropriate to the research question | | | The research evidence is of sufficiently high quality |
| • systematic reviews | • seen as one part of a decision | | | The research will be used as one part of the evidence |
| • evidenced based guidelines | • lack of certainty acknowledged | | | I value the research evidence |
|  | • social construction acknowledged | | | The research evidence fits with my understanding of the issue |
|  | • judged as relevant | | | The research evidence is useful in thinking about the issue |
|  | • importance weighted | | | I am clear about what the key messages for the planned intervention are |
|  | • conclusions drawn | | | There is consensus amongst my colleagues about the usefulness of this research to this issue |
| **Clinical Experience - sub-element of evidence** | | | | |
| • high levels of consensus | • clinical experience and expertise reflected upon, tested by individuals and groups | | | I have reflected on my own clinical experience in relation to this issue |
| • consistency of view | • consensus within similar groups | | | I have shared and critically reviewed my clinical experience in relation to this issue |
|  | • valued as evidence | | | I have shared and critically reviewed my clinical experience with knowledgeable colleagues outside of my (clinical) workplace |
|  | • seen as one part of the decision | | | There is a consensus of (clinical) experience about this issue |
|  | • judged as relevant | | | Clinical experience will be used as one part of the evidence |
|  | • importance weighted | | | The consensus of clinical experience fits with my understanding of the issue |
|  | • conclusions drawn | | | Clinical experience evidence is useful in thinking about the issue |
|  |  | | | I am clear what the key messages for the planned intervention are |
| **Patient Experience – sub-element of evidence** | | | | |
| • partnerships | • valued as evidence | | | We routinely (and systematically) collect users/patients’ experiences about this particular issue |
|  | • multiple biographies used | | | Users/patients experiences will be used as one part of the evidence |
|  | • partnerships with health care professionals | | | I value patient experiences evidence |
|  | • seen as one part of a decision | | The evidence of patients experiences fits my understanding of the issue(s) | |
|  | • importance weighed | | Patient experiences are useful in thinking about the issue | |
|  | • conclusions drawn | | I am clear about what the key messages for the planned intervention are | |
|  |  | | There is a consensus amongst my colleagues about the usefulness of patient experiences to this issue | |
| **Information/Data from Local Context – sub-element of evidence (new in phase 2)** | | | | |
| Not identified | • valued as evidence | | Data/information is routinely (and systematically) collected about this issue | |
|  | • collected and analysed systematically and rigorously | | Data/information from the local context will be used as one part of the evidence | |
|  | • evaluated and reflected upon | | I value the data/information from the local context | |
|  | • conclusions drawn | | The data/information from the local context fits with my understanding of the issue(s) | |
|  |  | | The date/information from the local context is useful in thinking about the issue | |
|  |  | | I am clear about what the key messages for the planned intervention are | |
|  |  | | There is a consensus amongst my colleagues about the usefulness of the information/data from the local context for this issue | |
| **CONTEXT: The environment or setting in which the proposed change is to be implemented** | **CONTEXT: dimensions viewed to be critical to successful implementation within the setting or environment where the new evidence was being implemented** | | **CONTEXT: specific questions derived from the dimensions to diagnose the “readiness” of the context for successful implementation and to evaluate the impact of the change using the same questions** | |
| Receptive Context – sub-element of context (new in phase 2) | | | | |
| Not identified | • physical / social / cultural / structural / system – boundaries clearly defined and acknowledged | | The physical location is conducive to the implementation of this issue | |
|  | • professional/social networks clearly defined and acknowledged | | I have access to the appropriate/useful professional networks and implement this intervention successfully | |
|  | • appropriate and transparent decision making processes | | Decision making processes in the organisation are clear to me | |
|  | • power and authority processes | | I have the power and authority to carry out this intervention | |
|  | • human/financial /technological/ equipment – resources appropriately allocated | | There are sufficient human resources to implement this intervention successfully  There is the right equipment to implement this intervention successfully  There are sufficient financial resources to implement this intervention successfully | |
|  | • information and feedback systems in place | | There is the right IT support to implement this intervention successfully | |
|  | • initiative fits with strategic goals and is seen as a key priority | | The intervention fits with the strategic intent and goals of the organisation | |
|  | • receptiveness/openness to change/new ideas | | I have access to the appropriate skills and knowledge to carry out this intervention | |
| **Culture – sub-element of context** | | | | |
| • learning organisation | • able to define culture(s) in terms of prevailing values/beliefs | | This organisation values innovation | |
| • patient centred | • values individual staff and clients | | This organisation values people who innovate | |
| • valuing people | • promotes learning of organisation | | This organisation values staff as individuals | |
| • continuing education | • consistency of individual role/experience to value:  - relationships with others  - team work  - power and authority  - rewards/recognition | | This organisation values open communication and dialogue | |
|  |  | | I feel there is open communication and dialogue within my immediate work place | |
|  |  | | I value open communication and dialogue | |
|  |  | | This organisation values collaborative partnership working | |
|  |  | | I feel there is collaborative partnership working in the wider organisation | |
|  |  | | I feel there is collaborative partnership working within my immediate work place | |
|  |  | | I value collaborative partnership working | |
|  |  | | There is a culture of continuous improvement in this organisation | |
|  |  | | There is a culture of continuous improvement with my immediate workplace | |
|  |  | | This organisation embraces change | |
|  |  | | This organisation values patients as individuals | |
|  |  | | My immediate workplace embraces change | |
|  |  | | This organisation involved key stakeholders when introducing change | |
| **Leadership –sub-element of context** | | | | |
| • clear roles | • transformational leadership | | I work within an effective team | |
| • effective teamwork | • role clarity | | I am clear what my role is within the team | |
| • effective organisational structure | • effective teamwork | | I am clear what my role is in the implementation of this initiative | |
| • clear leadership | • effective organisational structures | | I am clear what the lines of accountability are in terms of my role in implementing this initiative | |
|  | • democratic, inclusive decision making | | I have been involved in determining how this initiative is going to be implemented | |
|  | • enabling/empowering approach to learning / teaching/managing | | I have been able to develop new skills through this process  I feel that I have learnt new skills and competencies | |
| MEASURE:  Sub-element of context | **EVALUATION:**  **Term changed to**  **evaluation** | | **EVALUATION:** | |
| • internal measures used routinely | | • feedback on individual/team/system - performance | We have routine mechanisms in place to collect data on: | |
|  |  |  | • individual performance (e.g. appraisal, clinical supervision, 360° feedback) | |
|  |  |  | • team performance (e.g. audit and feedback, patient feedback, 360° feedback) | |
|  |  |  | • system performance (e.g. audit and feedback, formal inspections, economic data) | |
| • audit or feedback used routinely | | • use of multiple sources of information on performance | Multiple sources of evaluation are used routinely in my workplace | |
| • peer review | | • use of multiple methods  - clinical (o/c) / individual /personal/economic/(patient) experience - evaluations | This type of evaluative information is routinely used to improve and change practice | |
| • external measures | | Range of routine measures collected by teams | The external data we collect is used by us to inform and improve our everyday practice. | |
| Main ElementFACILITATION | | | | |
| A technique by which one person makes things easier for others  [4] | Facilitation refers to the process of enabling (making easier) the implementation of evidence into practice. | | Facilitation is a process that depends upon the person (the facilitator) carrying out the role with the appropriate skills, personal attributes and knowledge | |
| Characteristics (of facilitators)  • respect  • empathy  • authenticity  • credibility Role • access  • authority  • change agenda  • successfully negotiated Style • range and flexibility of style  • consistency and appropriate presence and support | It is achieved by an individual carrying out a specific role (a facilitator) which aims to help others. This suggests that facilitators are individuals with the appropriate roles, skills and knowledge to help individuals, teams and organisations apply evidence into practice  [6] | | The purpose of facilitation varies from providing help and support to achieve a goal to enabling individuals and teams to analyse, reflect and change their own attitudes, behaviours and ways of working  Facilitators gain experience through guided support and mentorship from expert facilitators. There are a set of key facilitation competencies that can be used to help develop the “enabling and empowering” role of the facilitator. | |
| Purpose **Role**  **Skills and attributes** | • appropriate mechanisms for facilitation in place TASK • doing for others:  - episodic contact  - practical/technical help  - didactic, traditional approach to teaching  - external agents  - low intensity – extensive coverage  • Task/doing for others:  - project management skills  - technical skills  - marketing skills  - subject/technical/ clinical credibility | | HOLISTIC • enabling others  - sustained partnerships  - developmental  - adult learning approach to teaching  - internal/external agents  - high intensity – limited coverage  • holistic/enabling  - co-counselling  - critical reflection  - giving meaning  - flexibility of role  - realness/authenticity | |
